# Supplementary material for: More coral, more fish? Contrasting snapshots from a remote Pacific atoll
Source: PeerJ. 2015 Jan 29;3:e745. doi: 10.7717/peerj.745 (PMC4314088; doi:10.7717/peerj.745)
Supplement: Table S1 — The numerator shows abundance in 1981, as reported by Bell & Galzin (1984); the denominator shows abundance in 2012, derived from the same surveying method (i.e., one 50-m transect) at the same sites. Zeros are omitted for clarity. Asterisks denote species considered to be reliant on live coral for shelter or food. [file peerj-03-745-s001.docx]

Table S1. Reef fish species, and their abundances (number of individuals per 250 m^2^), recorded at 13 sites in the lagoon of Mataiva Atoll, French Polynesia. The numerator shows abundance in 1981, as reported by Bell & Galzin (1984); the denominator shows abundance in 2012, derived from the same surveying method (i.e., one 50-m transect) at the same sites. Zeros are omitted for clarity. Asterisks denote species considered to be reliant on live coral for shelter or food.

Site

Taxon 1 2 3 4 5 6 7 8 9 10 11 12 13

**Synodontidae**

*Saurida gracilis* / / / / / / / / / / /1.5 / /

**Muraenidae**

*Gymnothorax buroensis* / / / / / / / / / /0.5 / / /

*G. javanicus* / / / / / /0.5 / /1 / / / 1/ /

**Holocentridae**

*Myripristis violacea** / / / / / / / / /0.5 / / / /

*Neoniphon argenteus* / / / / /2 / / /3.5 / /3 /2 /0.5 /

*N. sammara** / /1 / / / / / 1/ /1.5 / / / /

*Sargocentron spiniferum** / / / / / /1 / / /2.5 / /1.5 / /

**Serranidae**

*Cephalopholis argus** / / / / / / / / / / /0.5 2/1.5 /

*Epinephelus polyphekadion** / 1/ / / 1/ / / / / / / / /

**Apogonidae**

*Cheilodipterus macrodon* / /0.5 / / / / / /2 /0.5 /0.5 /1 / /

*C. quinquelineatus** 1/0.5 2/6.5 1/1.5 / 1/5 1/1.5 2/ 2/14.5 4/5 1/12.5 5/2 3/0.5 /0.5

*Pristiapogon exostigma* / / / / / / 1/ / / / / /1.5 /

*Ostorhinchus novemfasciatus* / / / / / / / / / / / 1/ /

**Carangidae**

*Caranx melampygus* / / / / / / / /0.5 1/ /0.5 / / /

**Lutjanidae**

*Lutjanus fulvus* / /1 1/1.5 2/3 3/1.5 12/1.5 /1.5 / 1/ 4/1.5 / 1/1 /

**Mullidae**

*Mulloidichthys flavolineatus* / 1/ / 1/ 6/1.5 /1 4/ /0.5 /3 / / / /17.5

*M. vanicolensis*  / / / / / /4 / / / / / / /

*Parupeneus insularis* / / / / / / / / / / / / 1/

*P. multifasciatus* / / / / / /0.5 / / /0.5 3/ /1 / 5/2.5

**Lethrinidae**

*Monotaxis grandoculis* / / / / / / / / / / / / /0.5

**Chaetodontidae**

*Chaetodon auriga** /1 1/ / /2.5 5/1 /1.5 /0.5 /5 1/5.5 /2.5 1/4.5 1/ 6/10

*C. bennetti** / / / / / / / /1 2/ /0.5 / / /

*C. citrinellus* / / / / / / / / / / / / 3/

*C. ephippium** /1.5 1/ 1/ /1.5 2/ 1/1 / 1/6 2/3.5 9/2.5 2/2.5 4/2.5 2/8

*C. lunula* / /0.5 / /3 1/ 1/ 1/ 1/ 2/9 / /1.5 / 10/

*C. lunulatus** /2.5 / /2 /7.5 / /1 /0.5 2/6.5 /11.5 16/3 4/7 /1.5 8/13

*C. semeion** / / / / / / / / / / /1 / /

*C. trifascialis** / / / / / / / / /1.5 / / / /

*C. ulietensis** / / / /1 1/ /0.5 / /1.5 /9 1/0.5 1/9 / 6/7.5

*C. unimaculatus** / / / / / / / / / / / / 1/

*C. vagabundus* / / / / / / / /1.5 / 1/ / / /12.5

**Pomacanthidae**

*Centropyge flavissima** /0.5 / / / / / / / / / 1/ / /

**Pomacentridae**

*Abudefduf septemfasciatus* / / / / /0.5 / / / / / / / /

*A. sexfasciatus*  / / /0.5 /0.5 /2 / /1 1/5 /1.5 /1 /0.5 /1 /

*Chromis margaritifer* / / / / / / / / / /0.5 / / /

*C. viridis** 7/2.5 15/1 /25 / / 27/72.5 56/ 27/135 92/ 30/6 24/0.5 69/103 /99

*Chrysiptera brownriggii* /1.5 / / /0.5 / / / / / / / 1/1.5 /

*Dascyllus aruanus** /38.5 / /68.5 /0.5 / 2/11.5 3/3 1/24.5 9/5.5 17/9.5 15/0.5 40/52.5 7/87.5

*D. flavicaudus*  / / / / / / / / / / / / /1

*Plectroglyphidodon* /0.5 / /1 / / / / /0.5 / / / / /

*phoenixensis**

*Pomacentrus coelestis* / / / / / 1/ 8/ 1/2 11/ / 11/ /3 /

*Stegastes nigricans** /66.5 / /42.5 / / / / / / / 3/0.5 1/28 15/1.5

**Labridae**

*Bodianus axillaris* / / / /0.5 / / / / / / / / /

*Bodianus* sp. / / / / / / / / / 1/ / / /

*Cheilinus trilobatus** /0.5 / / / / / / /0.5 / /0.5 / / 1/

*Cheilinus* sp. / / / / / / / / / / / 1/ /

*Coris aygula* / / / /1.5 / / / / / / / / /

*Coris gaimard*  / / / / 1/ / / / / / / / /

*Epibulus insidiator** / /0.5 / /3 / / /0.5 /4.5 /0.5 / /0.5 / /

*Gomphosus varius** / / / /5.5 / /0.5 / /2.5 / / /4 / 4/2.5

*Halichoeres hortulanus*  / / / / / / / /1.5 / / 1/0.5 20/ 5/

*H. marginatus** / / / / / / / 1/ / / / / /

*H. trimaculatus* /6 / /12.5 /1 /1.5 /0.5 / /2.5 /0.5 /1.5 /1 /14 /10

*Halichoeres* sp. / / / / / / / / / / / 3/ /

*Labroides dimidiatus** / / / / / / / / / / / /1 1/3

*Pseudocheilinus octotaenia* /0.5 / / / / / / / / / / 1/ 1/

*Stethojulis bandanensis* /0.5 / / /1 / / /0.5 /0.5 / /1 /2 /0.5 1/1.5

*Thalassoma amblycephalum* / / /2 /0.5 / / / /1.5 / / /0.5 3/11 1/

*T. hardwicke* /3.5 / /4.5 /8.5 /4.5 /2.5 /1.5 /13 1/9 2/5 2/20.5 3/2 5/15

*T. quinquevittatum* /1 / / /8.5 / /3 /2 /4.5 /0.5 /1 4/12 / 2/1.5

*Wetmorella* sp. / / / / / / / / / / / 1/ /

Unidentified labrid / / / / / / / / / / / / 1/

**Scaridae**

*Chlorurus sordidus* /28.5 /34 /40.5 /119 5/11 /25 5/20 2/75 1/76 65/57.5 9/93.5 7/13.5 34/70.5

*Hipposcarus longiceps* / / / / 2/ / /0.5 / /1 / / / 3/

*Scarus ghobban* / / / / 2/ 1/ / / / 2/ / / 2/

*S. oviceps* /0.5 / / / / / / / / 2/ /0.5 / /

*Scarus* sp. 1/4.5 1/2 2/6 1/14 24/1.5 /1 1/1 8/14.5 4/2.5 /6.5 11/2 53/4 6/12.5

**Acanthuridae**

*Acanthurus triostegus* /8.5 /0.5 1/3.5 1/38 5/8 /5.5 /10 1/26 2/8 14/11 15/4 6/4 20/3

*Ctenochaetus striatus* /0.5 / / /0.5 / / / /2 / 1/ /1 /3 11/

*Zebrasoma scopas** / / / /0.5 / / / / /0.5 / / / /

*Z. velifer* / / / /0.5 3/ /0.5 / /3 /2.5 2/ 1/ / 4/

**Gobiidae**

*Amblygobius nocturnus* 4/1.5 2/1 5/ 1/ / / 2/ /1 / / 1/0.5 / /0.5

*A. phalaena* 4/5.5 11/5 14/9 3/5 2/16 4/4.5 9/4.5 16/12 13/5.5 1/23 35/11 13/4 8/8

*Asterropteryx semipunctata* / / / / / / / / 1/ / / 7/ 20/

*Ctenogobiops feroculus* /1.5 /1.5 / /1.5 / /0.5 /1.5 / /1 /9.5 /1.5 / /

Eviota spp. / /1 /1.5 /0.5 /6.5 /2.5 /0.5 / / /2.5 / / /

*Gnatholepis anjerensis* / / / / / / / / / / / /1 /

*Valenciennea strigata* / / / / / / / / / /1 / / /

*Unidentified goby* /1 /1.5 / /0.5 / / /1 /2.5 / /3.5 / /10.5 /5.5

**Blenniidae**

*Blenniella periophthalmus* / / / / / / / / / / / 1/1 /

*Ecsenius* sp. / /1.5 / /0.5 /0.5 /0.5 /0.5 /0.5 / / / /0.5 /0.5

**Bothidae**

*Bothus mancus* / / / / / / 1/ / / / / / /

**Balistidae**

*Rhinecanthus aculeatus* / / / / / / / / / / 1/ 1/ 1/

**Ephippidae**

*Platax* sp. / / / / / / / / /0.5 /2 / / /

**Tetraodontidae**

*Canthigaster bennetti* / / / / / / / / / / / 1/ 4/

*C. solandri* / / / 0/1 1/ 1/1 /1.5 / 1/1 / 1/1 1/ /6

**Ostraciidae**

*Ostracion cubicus** / / / / / / / / / / 1/ / /
