# Supplementary material for: More coral, more fish? Contrasting snapshots from a remote Pacific atoll
Source: PeerJ. 2015 Jan 29;3:e745. doi: 10.7717/peerj.745 (PMC4314088; doi:10.7717/peerj.745)
Supplement: Table S2 [file peerj-03-745-s002.docx]

Table S2. Proportion of reef fish species which were present in the Mataiva lagoon in1981 but not in 2012, and vice versa, in relation to trophic group.

Herbivores Coralivores Invertivores Piscivores Planktivores

Present in 1981, not 2012 23% 15.5% 46% 15.5% 0%

Present in 2012, not 1981 23% 6% 47% 6% 18%

Table S3. Comparison of linear and asymptotic models of fish richness and abundance with coral cover 1981-2012.

Linear models, y = b*x + a, were fit using the lm() function in R. Asymptotic models were fit using the Michaelis-Menten equation, y =( a*x)/(b+x), with the nls() function in R. AICc values were compared using the model.sel function in the R package MuMIn.

‘Single transect’ means that the 2012 data are derived from a single transect, to mimic the survey design of Bell and Galzin (1983). ‘Multiple transects’ means that the 2012 data are derived from the average of three transects.

The parameters a and b, their standard errors and AICc values are given for each model. The number of parameters estimated k = 3 in all models. Δ AICc values are reported for each comparison as the deviation from the best supported model, which is indicated in bold. Δ AICc values > 2 indicate substantially stronger support for the model in bold.

a ± se b±se AICc ΔAICc

*Single transect*

(a) Fish species richness

Linear 15.03 ± 2.11 0.31 ± 0.09 183.9 11.32

**Asymptotic 27.80 ± 2.30 1.91 ± 0.89 172.6 0**

(b) Fish abundance

Linear 316.3 ± 26.90 2.82 ± 1.17 316.3 8.01

**Asymptotic 216.50 ± 33.41 2.42 ± 1.91 308.2 0**

*Multiple transects*

(c) Fish species richness

Linear 14.11 ± 2.00 0.22 ± 0.09 179.0 7.95

**Asymptotic 22.72 ± 1.95 1.18 ± 0.73 171.1 0**

(d) Fish abundance

Linear 97.46 ± 27.58 3.15 ± 1.19 315.5 9.15

**Asymptotic 225.81 ± 31.79 1.91 ± 0.89 306.4 0**
